# The patient perspective on transanal irrigation treatment for low anterior resection syndrome after rectal cancer surgery - a qualitative and quantitative study

**DOI:** 10.1186/s12876-025-03633-4

**Published:** 2025-02-07

**Authors:** Boglarka Rethy, Anna Schandl, Caroline Nordenvall, Gabriella Jansson Palmer, Charlotta Bergström, Maria Williamson, Emil Pieniowski, Asif Johar, Pernilla Lagergren, Mirna Abraham-Nordling

**Affiliations:** 1 Department of Molecular Medicine and Surgery, Karolinska Institutet, Stockholm, Sweden; 2 Department of Perioperative- and Intensive Care, South General Hospital, Stockholm, Sweden; 3 Department of Pelvic Cancer, Division of Coloproctology, Center for Digestive Diseases, Karolinska University Hospital, Stockholm, Sweden; 4 Department of Surgery and Urology, Danderyd Hospital, Stockholm, Sweden; 5 Department of Women´s and Children´s Health, Karolinska Institutet, Stockholm, Sweden; 6 Department of Surgery, South General Hospital, Stockholm, Sweden; 7 Department of Surgery and Cancer, Imperial College London, London, UK

**Keywords:** Cancer survivorship, Bowel dysfunction, Follow-up, Patient experience

## Abstract

**Purpose:**

The aim of the study was to explore long-term experiences of transanal irrigation (TAI) in patients with major low anterior resection syndrome (LARS).

**Methods:**

The study included a qualitative and quantitative analysis of patients who developed major LARS after rectal cancer surgery between 2016 and 2019 and have undergone treatment with TAI. The patients received questionnaires. Mean scores were calculated with time-to-deterioration. Individual semi-structured interviews were performed and analyzed, according to Graneheim and Lundman with patients who performed TAI regularly for more than one year.

**Results:**

In total 28 out of 39 patients responded to the questionnaires and 16 patients participated in the interviews. At mean 6-years follow-up, a 9.4 points difference in mean LARS score was obtained, (21.2 vs. 30.7) indicating less LARS symptoms in favor of the TAI treatment. Patients in the TAI group used less loperamide compared to the control group (36% vs. 79%). The use of bulky agents was similar. The interview text rendered into three main categories: *regaining control in everyday life*, *need for structure and planning and becoming familiar with the procedure.*

**Conclusions:**

Treatment with TAI showed the potential to improve the quality of life of patients with major LARS. The improvements in their general well-being were valued over adjustments and time spent on TAI.

**Implications for cancer survivors:**

Bowel dysfunction remains after 6-years with lower LARS scores favoring the TAI treatment. In the absence of a definitive treatment, survivors of rectal cancer coping with LARS have shown appreciation of the TAI treatment.

**Supplementary Information:**

The online version contains supplementary material available at 10.1186/s12876-025-03633-4.

## Introduction

The change in surgical strategy in treatment for rectal cancer, from abdominoperineal resection to low anterior resection (LAR) with sphincter preservation has led to long-term dysfunctional side effects, known as low anterior resection syndrome (LARS) [1, 2]. The prevalence of LARS varies between 60 and 80% in patients undergoing LAR [3]. Major LARS occurs in 18–53% and is associated with impaired quality of life (QoL) [4–7]. Treatment of LARS aims to reduce symptoms in the absence of a definitive treatment. A previous randomized controlled trial from this group revealed that patients with major LARS randomized to transanal irrigation (TAI) experienced reduced LARS-symptoms and improved QoL after 12 months compared with the conservatively treated group [8]. TAI was performed at home with an irrigation system using both air and water to regulate irrigation of the colon/neorectum.

Long-term follow-up evaluations of TAI as a treatment of LARS are scarce. Rosen et al. evaluated patients with TAI treatment after 12 months and found that only approximately half of the patients (10 out of 19) continued with the treatment, even though they had lower LARS scores compared with the control group [9]. There are numerous reasons as to why patients do not continue with the treatment, as, improvement of LARS symptoms over time, patients coping with the symptoms or compliance with the TAI procedure decreases during follow-up [10–12].

A small qualitative study carried out 6 months after start with TAI reported that TAI was considered an acceptable treatment method for LARS. The patients with severe symptoms were more likely to consider rectal irrigation as a treatment option [13]. The long-term effects of TAI on bowel function, QoL and compliance with the treatment have not been investigated over time.

The aim of the study was to investigate LARS over time and to explore patients’ experiences with TAI, bowel function, QoL and evaluate compliance to TAI, as a follow-up of patients within a previous TAI randomized controlled trial (RCT) [8].

## Methods

### Study population and design

This is a multicenter follow-up study with combined qualitative and quantitative methodologies including patients from a previous randomized control study (RCT) [8]. Patients had undergone surgery for rectal cancer with total mesorectal excision (TME) and a defunctioning ileostomy between May 2016 and November 2019 in Stockholm County, Sweden [8]. Written consent was obtained from the patients.

### Questionnaires

Eligible patients from both the intervention (TAI) and control (conservative) groups were requested to answer four questionnaires (same as in the original study) [8]. Three questionnaires were sent regarding bowel function; the LARS score questionnaire [6, 14], Cleveland Clinic Florida Fecal Incontinence Score (CCFFIS) [15] and four-study specific questions regarding bowel function *(“How would you describe your bowel function in general?”*,* “How does your bowel function affect your daily life?”*,* “If you have trouble with urgency*,* how does this affect your daily life?”*,* “If you have trouble with fragmentation*,* how does this affect your daily life?”*,* [all questions graded 1–10*,* 1 = not at all*,* 10 = very negative*,* 0 = no experience])* [8] and one health-related QoL questionnaire European Organization for the Research and Treatment of Cancer Quality of Life Questionnaire Core 30 (EORTC QLQ-C30) [16, 17].

All patients were asked the use of bowel-related medication and patients in the TAI group answered questions regarding TAI.

The data (interviews and questionnaires) of the present study were collected at mean follow-up time 6-years after the last follow-up in the original study.

### Statistical analysis

The mean scores for LARS, the CCFFIS, the 4 study-specific questions and the EORTC QLQ-C30 subscales/items were calculated with time-to-deterioration. The mean scores from the previous follow-up measurements were re-calculated using the same method [8]. For patient characteristics descriptive statistics were generated for all baseline demographic and clinical characteristics. Continuous variables were summarized using means and standard deviations, while categorical variables were presented as frequencies and percentages.

To account for repeated measures and adjust for baseline LARS score, a mixed-effects regression model was employed using the PROC MIXED procedure, with time as a repeated factor and subject-level variability controlled by including patient as a random effect. All statistical tests were two-tailed, with 5% level of significance. Statistical analyses were performed using SAS software (version 9.4, SAS Institute Inc., Cary, NC, USA).

### Interviews

#### Data collection

Individual interviews were held with patients in the TAI group and also included those in the control group who had started TAI after the end of the trial. These patients had previously received education in performing transanal irrigation by a urotherapist or a stoma nurse and had received continuous prescriptions of the Peristeen device [8].

A semi-structured interview guide was used that included ten questions regarding different experiences with TAI and its influence on daily life (Supplementary Material, SM1). Two nurses (CB, MW) and two physicians (BR and MAN) conducted the interviews. The patients were divided equally between the two groups based on which hospital they belonged to. The participants had no previous relation to the interviewers. Two pilot interviews were carried out, one by each group, to evaluate the relevance of the questions. As no adjustments to the interview guide were made the pilot interviews were included in the analysis. Patients could choose between telephone-based or in-person interviews at the hospital.

#### Data analysis

The interviews were recorded and transcribed verbatim. The data was analyzed according to inductive qualitative content analysis, as described by Graneheim and Lundman [18]. The research team members had great knowledge of the patient group and TAI. The research members AS and PL were well experienced with the qualitative method design and worked in close collaboration with the members interviewing the patients. The interview transcripts were read by all researchers to become familiar with and gain mutual understanding of the content. Four researchers (BR, CB, MAN and MW) continued the process identifying meaning units that answered the aim of the study. The units were carefully discussed and compared to settle on code application. One member (BR) continued the coding and had close discussion with the other members throughout the process. Internal discussions followed about resemblance and disparities among the codes. The codes were organized into subcategories and categories after consensus among the members. The achieved analysis was reflected on, compared with quotations and the original interviews. The titled subcategories and categories were adjusted until full consensus was obtained among the researchers. See the example of the analysis process (Table 1).

**Table 1:** Analysis process

Main Categories	Subcategories	Codes	Meaning Units
Regaining Control in Everyday Life	Regaining Self-Control	Freedom	*”… I would not have been able to visit my friends*,* I nearly couldn´t go to a store because of it*,* that I always needed to go to the toilet…” (Patient 4)*
		Control	*”… I can control my own situation much better with this than when you try with other methods.” (Patient 3)*
	Regularity for Effect	Effect	*” Yes*,* I think this works*,* from all I have heard*,* read and seen this must be the cleanest*,* easiest and most effective system*,* I think.” (Patient 10)*
	Physical Symptoms	Abdominal Pain	*“… I could feel it a little*,* but it wasn´t that bad*,* it was more of a discomfort…” (Patient 8)*
Need for Structure and Planning	Planning of the Procedure	Planning	*”…it takes some balancing act*,* you are more secure with TAI*,* if I think about the nights…then I do it before and then I am 90% sure I will survive the night…” (Patient 10)*
	Routine Use	Easy	*”…it was like knotting the shoes or it was very easy and less complicated than what I thought the experience would be like.” (Patient 2)*
	Time	More Time	*“…It has given me many hours back and it has made it possible for me to be more free with what I do.” (Patient 10)*
Becoming Familiar with the Procedure	Tool	Material Dependence	*”…you can always take it with you*,* when out canoeing or to the cottage/lake house*,* it is possible to bring it along anywhere.” (Patient 1)*
	Procedure Discomfort was Reduced by Training	Anxiety for Damage	*”…I can say that when it hurt a little and so*,* then I got a bit anxious that it would not be good for the bowel.” (Patient 4)*

### Ethical approval

The study was approved by the Local Ethics Committee of Stockholm (2023-01438-02, 2017/551 − 31 and 2019–04398).

## Results

**Fig. 1:**
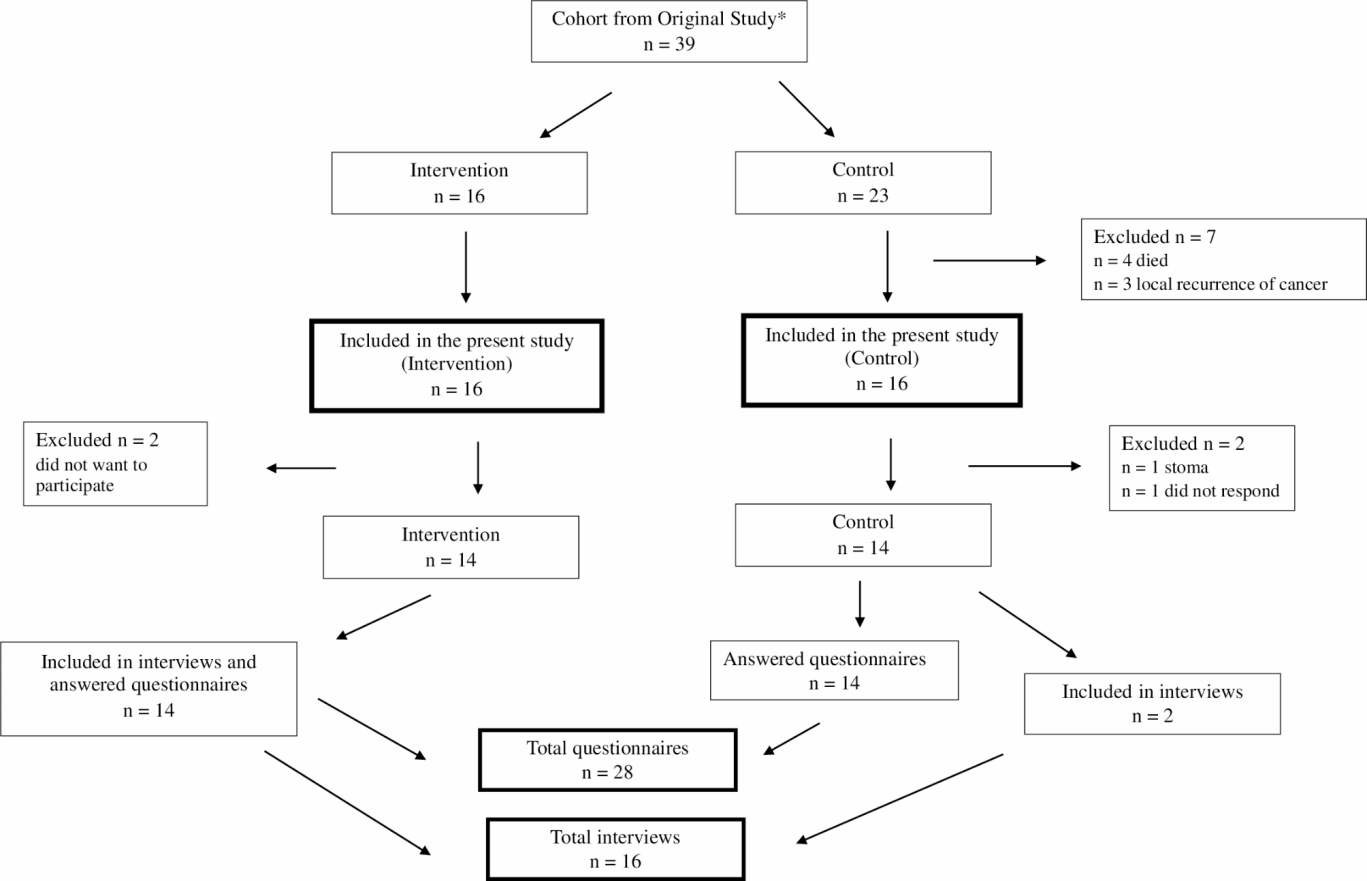
Flow chart. *Reference 6

In total, 28 out of 39 patients were included in the final quantitative analysis, out of which 16 patients were included in the qualitative analysis (Fig. 1). Two patients from the control group started with TAI after the previous study ended. They remained in the control group in the quantitative analysis but were included in the qualitative analysis as they performed TAI. The mean age at primary surgery was respectively 64 years and 62 years for the TAI and control groups. There were no patients with anastomotic leakage. In both groups 5 patients received neoadjuvant radio/chemotherapy. Mean time to stoma reversal was longer for the TAI group, 212 days vs. 190 days. The mean follow-up time was 5.80 years (1.03, standard deviation [SD]) for the entire cohort, 6.04 years (0.90 SD) for the TAI group and 5.56 years (1.12 SD) for the control group (Table 2). The responses to the questionnaires from the previous study baseline (= 0 month), 6 months and 1-year were added to the results from this follow-up study.

**Table 2:** Patient characteristics for intervention (TAI) and control groups

	TAI *n* = 14 (%)	Control *n* = 14 (%)
Age at primary surgery mean, (year)*	64 (9.8)	62.2 (9.3)
Sex, male/female	9 (64) /5 (36)	8 (57) /6 (43)
BMI mean*	25.8 (4.1)	27.6 (4.4)
Tumor level, (cm)*	10.2 (1.7)	10.1 (2.5)
Preoperative T-stage > 3	10 (71)	11 (79)
Preoperative N-stage > 1	11 (79)	9 (64)
Neoadjuvant radiotherapy	5 (38)	5 (38)
Neoadjuvant chemoradiotherapy	5 (38)	5 (38)
Surgical approach		
Open	4 (29)	4 (29)
Minimally invasive approach Laparoscopic Robotic Conversion	10 (71) 1 (7) 9 (64) 0 (0)	12 (86) 2 (14) 8 (57) 2 (14)
Type of anastomosis		
End to end Side to end	0 (0) 14 (100)	1 (7) 13 (93)
Pathology T-stage > 3	5 (38)	5 (38)
Pathology N-stage > 1	7 (50)	5 (38)
Adjuvant chemotherapy	4 (29)	2 14)
Anastomotic leakage	0 (0)	0 (0)
Time to stoma reversal mean, (days)*	212 (91)	190 (76)
Time to follow-up mean, (year)*	6.04 (0.90)	5.56 (1.12)

*Standard deviation

### Questionnaires

#### Mean scores and differences between TAI and control groups in LARS Score, CCFFIS and 4-study-specific questions

The mean LARS scores were similar in the TAI and control groups at study entry. The LARS score decreased significantly during the first 6 months in the TAI group (36.4 to 20.4 vs. 35.7 to 31.6). This difference − 9.4 (95% CI: -16.2 to -2.8), *p* = 0.0068 remained at 6-years of follow-up 21.2 vs. 30.7 in TAI and control groups respectively. CCFFIS showed no significant differences between the two groups. In the four-study specific questions, statistically significant difference in mean score, -1.6 was seen at 6 months in question 1 (*“How would you describe your bowel function in general?”* and in question 3 and 4 (*“If you have trouble with urgency*,* how does this affect your daily life?” and “If you have trouble with fragmentation*,* how does this affect your daily life?”* -2.7 vs. 2.9 respectively at 6 months in favor for the TAI group (Table 3).

**Table 3:** Mean scores and differences between intervention (TAI) and control groups in LARS score, CCFFIS and the 4 study-specific questions

Outcome Measure	Group	0 month		6 months		1-year		6-years	
		Mean score	Difference in Mean Score (95% CI)	Mean score	Difference in Mean Score (95% CI*)	Mean score	Difference in Mean Score (95% CI*)	Mean score	Difference in Mean Score (95% CI*)
LARS score	TAI	36.4	0.7 (-1.4 to 2.8)	20.4	**-11.1 (-17.0 to -5.3)**	23.0	**-9.2 (-15.1 to -3.4)**	21.2	**-9.4 (-16.2 to -2.8)**
	Control	35.7		31.6		32.2		30.7	
CCFFIS	TAI	9.6	0.0 (-2.7 to 2.7)	6.2	-1.7 (-4.6 to 1.2)	6.5	-2.5 (-5.4 to 0.4)	5.9	-1.3 (-4.7 to 2.1)
	Control	9.6		7.9		9.0		7.3	
Question 1:	TAI	6.1	-0.4 (-1.6 to 0.7)	4.2	**-1.6 (-2.9 to -0.2)**	4.6	-0.9 (-2.2 to 0.5)	4.7	-0.7 -2.3 to 0.8)
	Control	6.5		5.7		5.5		5.5	
Question 2:	TAI	5.5	-0.4 (-1.8 to 0.9)	3.7	-1.3 (-2.8 to 0.3)	3.7	-1.5(-3.1 to 0.0)	4.4	-0.7 (-2.6 to 1.0)
	Control	5.9		4.9		5.3		5.2	
Question 3:	TAI	5.9	-0.3 (-1.8 to 1.2)	2.0	**-2.7 (-2.6 to -0.8)**	3.2	-1.3 (-3.2 to 0.6)	4.0	-0.2 (-2.4 to 2.0)
	Control	6.2		4.7		5.6		4.1	
Question 4:	TAI	6.3	-0.5 (-1.9 to 0.9)	2.4	**-2.9 (-4.8 to -1.0)**	2.8	**-2.6 (-4.5 to -0.8)**	2.9	-2.1 (-4.3 to 0.1)
	Control	6.8		5.3		5.5		5.0	

*Adjusted for baseline LARS

CCFFIS: Cleveland Clinic Florida Fecal Incontinence Score

CI: confidence interval

Statistically significant (*p* ≤ 0.05) mean score differences and CI in bold

#### EORCT QLQ-C30 mean scores and differences comparing TAI and control groups

At 6-years follow-up there were no statistically significant difference in mean summary score between the groups (85.7 vs. 84.1). In contrast at 1-year follow-up the TAI group reported a statistically significant better score. The patients’ estimated global health scores were highest at 6-years follow-up, although the greatest deviation between TAI and the control groups was seen after 1-year 17.3 (95% CI: 3.8 to 30.7) *p* = 0.0118. Among the individual functional scales (physical and role functioning) and symptoms scales (fatigue, pain and diarrhea) significant differences (both statistical and clinical relevance) were observed at 1-year but there were no significant differences after 6-years (Table 4).

**Table 4:** EORTC QLQ-C30 Mean scores and differences between intervention (TAI) and control groups

Subscale/item	Group	0 month		6 months		1-year		6-years	
		Mean score	Difference in Mean Score (95% CI)	Mean score	Difference in Mean Score (95% CI*)	Mean score	Difference in Mean Score (95% CI*)	Mean score	Difference in Mean Score (95% CI*)
Summary score	TAI	82.8	1.2 (-5.7 to 8.0)	90.2	6.2 (-1.7 to 14.1)	91.6	**10.3** ^**L**^ **(2.4 to 18.2)**	85.7	1.6 (-7.6 to 10.7)
	Control	81.6		84.0		81.3		84.1	
Global health status/QoL	TAI	70.1	2.3 (-9.4 to 14.0)	72.0	4.9 (-8.5 to 18.2)	80.4	**17.3** ^**L**^ **(3.8 to 30.7)**	81.1	12.6 (-3.0 to 28.1)
	Control	67.8		67.2		63.1		68.6	
Functional scales									
Physical functioning	TAI	87.9	0.3 (-8.6 to 9.2)	96.8	**10.5** ^**S**^ **(1.9 to 19.0)**	96.4	**10.9** ^**S**^ **(2.4 to 19.4)**	92.2	1.8 (-8.1 to 11.7)
	Control	87.5		86.3		85.5		90.5	
Role functioning	TAI	77.3	-2.4 (-14.6 to 9.7)	83.8	9.8 (-4.8 to 24.4)	92.8	**14.8** ^**S**^ **(0.2 to 29.4)**	92.9	15.6 (-1.3 to 32.6)
	Control	79.7		84.0		88.0		77.3	
Emotional functioning	TAI	82.6	-1.8 (-12.6 to 8.7)	84.6	-0.9 (-13.1 to 11.4)	86.7	7.6 (-4.6 to 19.9)	81.7	-4.1 (-18.3 to 10.1)
	Control	84.4		85.5		79.0		85.8	
Cognitive functioning	TAI	84.8	-1.4 (-12.5 to 9.8)	85.4	-1.0 (-13.0 to 10.9)	89.5	3.9 (-8.0 to 15.8)	79.7	-6.0(-19.9 to 7.8)
	Control	86.2		86.4		85.6		85.7	
Social functioning	TAI	78.0	2.7 (-10.2 to 15.5)	90.2	9.5 (-5.7 to 24.6)	89.1	14.5 (-0.7 to 29.6)	87.4	8.4 (-9.2 to 26.0)
	Control	75.4		80.7		74.7		79.0	
Symptoms scales									
Fatigue	TAI	22.2	-3.9 (-16.7 to 9.0)	14.1	12.8 (-27.8 to 2.2)	9.0	**-20.4** ^** L**^ **(-35.5 to -5.4)**	16.7	-8-8 (-26.2 to 8.7)
	Control	26.1		26.9		12.3		25.5	
Nausea and vomiting	TAI	3.0	-0.6 (-5.2 to 4.0)	1.8	0.8 (-3.1 to 4.8)	2.8	0.4 (-3.6 to 4.3)	4.5	1.7 (-2.8 to 6.3)
	Control	3.6		0.9		2.4		2.8	
Pain	TAI	12.1	-0.2 (-13.1 to 12.7)	6.0	-7.4 (-20.1 to 5.6)	3.9	**-14.0** ^**M**^ **(-27.0 to -1.0)**	14.8	1.1 (-4.0 to 16.2)
	Control	12.3		13.5		17.9		13.7	
Single items									
Dyspnea	TAI	18.2	6.6 (-8.0 to 21.1)	20.2	-1.1 (-16.9 to 14.8)	5.6	-11.0 (-26.9 to 4.9)	14.5	-3.3 (-21.7 to 15.1)
	Control	11.6		21.3		16.6		17.8	
Insomnia	TAI	19.7	0.9 (-13.1 to 14.9)	14.5	-8.0 (-25.0 to 8.9)	18.6	-0.8 (-17.7 to 16.2)	22.0	9.6 (-10.0 to 29.3)
	Control	18.8		22.5		19.4		12.4	
Appetite loss	TAI	4.5	-5.6 (-16.8 to 5.6)	3.4	1.4 (-6.2 to 9.1)	1.3	-5.2 (-12.8 to 2.4)	4.2	0.8 (-8.1 to 9.6)
	Control	10.1		2.0		6.5		3.4	
Constipation	TAI	30.3	-0.1 (-23.3 to 23.1)	17.2	-6.5 (26.8 to 13.8)	13.0	-16.7 (-3.9 to 3.6)	20.2	-5.7 (-29.2 to 17.8)
	Control	30.4		23.6		30.0		25.9	
Diarrhea	TAI	24.2	-14.9 (-33.1 to 3.4)	0.8	**-19.7** ^**M**^ **(-34.6 to -4.7)**	9.1	-14.3 (-29.3 to 0.7)	22.8	− 0.3 (-17.7 to 17.0
	Control	39.1		20.4		23.4		23.1	
Financial difficulties	TAI	1.5	-1.4 (-6.5 to 3.7)	0.5	-3.7 (-12.8 to 5.3)	2.6	− 3.2 (-12.3 to 5.9)	5.2	1.1 (-9.5 to 11.6)
	Control	2.9		4.2		5.8		4.0	

*Adjusted for baseline LARS score

CI: confidence interval (*p* ≤ 0.05), ^S, M and L^: small, medium or large clinical difference

Differences with both clinical relevance and statistical significance are presented in bold

The guidelines for interpretation of the EORTC QLQ-C30 were used. It did not present guidelines to interpret mean scores for summary score and emotional functioning, these were considered clinically relevant when ≥ 10 points. For the subscales diarrhea and financial difficulties medium difference was the highest grade

#### Comparison between TAI and control groups concerning medication related to bowel function

**Fig. 2:**
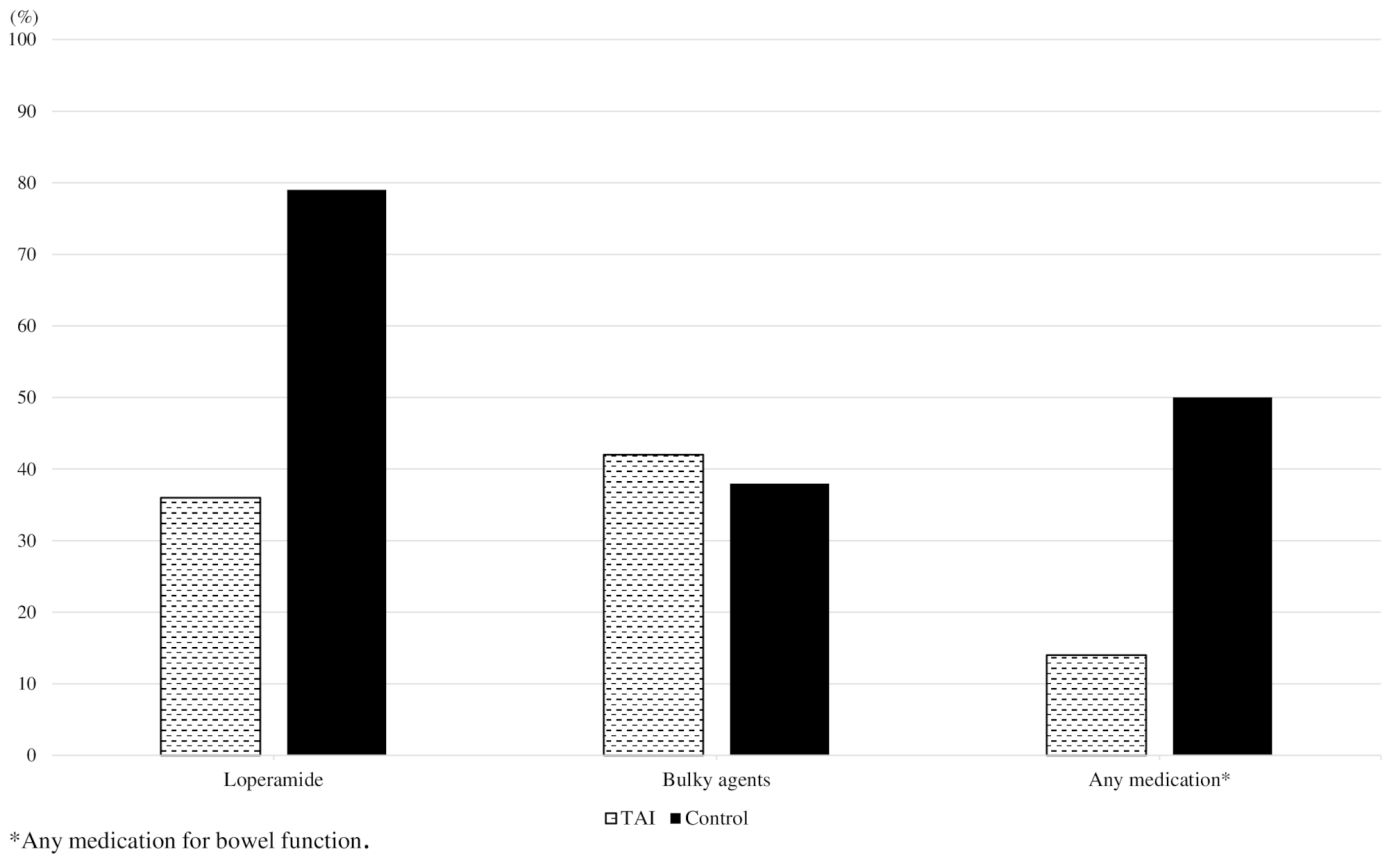
Comparison between Intervention (TAI) and Control groups concerning medication related to bowel function at mean 6-years follow-up

At the 6-years follow-up, patients in the TAI group used less loperamide than the control group, 36% vs. 79%, respectively. The consumption of bulky agents was relatively similar, 42% for the TAI and 36% for the control group. The prevalence of any bowel regulating medication was 50% vs. 92% in the TAI and control groups (Fig. 2).

### Interviews

There were three main categories identified in the qualitative analysis: *regaining control in everyday life*,* need for structure and planning and becoming familiar with the procedure*, all describing what role TAI had in different aspects of the patients’ lives (Table 1).

### Regaining control in everyday life

The qualitative study revealed that TAI was an important part of the patients` everyday lives and personal well-being. The effects of TAI resulted in a more consistent and reliable bowel function. Bowel movements were experienced more consistent and emptying more complete. Patients experienced two different effects when utilizing TAI. Bowel emptying was more regular that resulted in greater predictability and fluctuation decreased which positively influenced social contexts. The frequency of bowel emptying decreased and gave a sense of increased self-control on bowel habits. TAI was also considered as a mitigant to initiate bowel emptying. A patient even expressed it as the only option, exemplified by the following citation:

*“…for me it was the only way to empty the bowel.” (Patient 6)*.

The patients reflected that they felt less restricted in everyday life after being introduced to the TAI procedure, as they expressed improved control over their bowel function and an increased sense of self-determination over toilet visits. This resulted in the possibility to return to work and to participate in social activities as they were no longer continuously dependent on closeness to toilets. However, the patients described that their bowel dysfunction seemed to worsen when pausing the use of TAI. They perceived that TAI was necessary for a more balanced bowel function and emphasized the restored value of freedom allowing them to participate in activities. With TAI the patients experienced regained sense of self-control and no longer felt restricted in their activities. One patient even described TAI as part of life and not a treatment, exemplified by the following citation:

*“… I don´t see it is a treatment*,* you know it is part of life…” (Patient 7)*.

The patients described a reduction in embarrassment and increase in confidence when discussing their bowel dysfunction and the use of TAI with their surroundings. Previously viewed as a taboo, this openness alleviated psychological distress and anxiety associated with such a sensitive topic. Regarding other symptoms such as flatus, hick-ups and an urge to open the bowel, TAI was described to have different effects and they could remain the same, disappear or improve.

### Need for structure and planning

The need for structure and planning in everyday life describes how patients planned and fitted TAI in their daily routines, including the need for the correct device and place to be. The study revealed that performing TAI becomes a part of daily routine after being acquainted with the method. Patients found it to be an effective treatment and as an easy complementary solution when needed. Its straightforward nature made it easy to incorporate into their daily routine. One patient compared it to brushing their teeth, noting that it was less complicated than one might expect.

*“…it goes without problem 99 out of 100 times…” (Patient 2)*.

Given that the TAI procedure took 45 min to 1 h, experienced by the patients, planning was essential for them. Patients described the importance of performing TAI in a calm and stress-free environment with enough time allocated to ensure that, as it is not a short toilet visit. A patient described with the following citation how one should try to eliminate stress and be able to dedicate the necessary time:

*“…but you need to be prepared to allocate more time to perform in peace and quiet…” (Patient 1)*.

The additional planning and time management were described as providing a greater sense of control over the bowel function. With TAI, bowel emptying became more predictable and structured, in contrast to the unpredictability experienced without it. However, the intervals between each TAI procedure could lead to a feeling of being “locked in” until the next session. The patients described a need to take the possible intervals between toilet visits into consideration in daily planning. By doing so, TAI made them feel more secure in daily life even though it was not a complete solution.

### Becoming familiar with the procedure

The patients found that the effectiveness of TAI relied on using the correct materials and that they had to become accustomed to the process. The TAI-kit accompanied the patients during travels, while it was extra material to bring, it was considered to be an easy accessory to carry anywhere, even to less urbanized areas. Being familiar with the instruments and the procedure made it possible to easily perform in an everyday manner as exemplified in the following citation:

*“There were no issues…it felt a bit weird in the beginning*,* but you get used to it…” (Patient 9)*.

One negative aspect of TAI reported by the patients was pain or discomfort during or after the procedure. The pain could evoke anxiety and worry regarding damage to the bowel. However, these symptoms appeared to be random and could not always be linked to a specific technique or other factors even though the patients performed TAI regularly in a similar way. The pain and discomfort related to the device improved or completely disappeared after getting used to it. This is described in the following citation:

*“… it was not that bad but a little discomfort in the beginning perhaps…” (Patient 5)*.

## Discussion

This study aimed to investigate personal experience of the TAI treatment and long-term follow-up of bowel dysfunction and QoL in patients with major LARS following rectal cancer treatment. At mean 6-years of follow-up, the analysis showed significant differences in mean LARS scores (21.2 vs. 30.7 respectively), indicating less bowel symptoms in the TAI group, however no significant difference in QoL was revealed. The qualitative interviews identified aspects related to different life-related features associated to the use of TAI.

TAI seemed to require a new approach to planning and structuring daily life, which each patient had to adapt to in addition to adjusting to LARS itself after rectal cancer treatment [19]. This includes the need for a suitable environment and allocating time for TAI. In the present study, TAI resulted in regular bowel emptying with fewer unexpected defecation episodes, giving the patients more predictability in daily life. It was also revealed that a larger proportion of the patients in the TAI group were medication-free in comparison with the control group. This indicates that the TAI treatment alone could be enough to achieve a more regular bowel function. The incontinence score (CCFFIS) and the four-study specific questions, even though not statistically significant at 6-years follow-up, the observed difference at 6-months remained and were in line with these observations. Apart from incorporating TAI in a daily life, patients´ need to allocate time for hospital visits in the beginning to learn the TAI procedure. Thereafter the patients have continuous contact with health care practitioners for renewed prescriptions and if needed, guidance with the procedure.

*Burch et al.* report the difficulty of planning and being a part of social activities with LARS [20]. In the present study, the patients described that the use of TAI resulted in regained possibility to plan and to accustom their previous habits to their desire. This in line with another study showing a sense of regained freedom and security owing to regular defecations after using TAI [13]. Furthermore, the fear and the anxiety of unexpected bowel accidents decreased in most patients in the current study. Patients no longer avoided social activities nor were forced to carry extra bags with clothes or pads/wipes. However, the TAI equipment still accompanied them during travels, which indeed was an extra necessity but the reason behind this now was not fear, it was instead an option for more safety and more controlled bowel function.

During the interviews, the anxiety and the taboo around this sensitive matter were explored. As presented in the current literature the emotional burden and stigma of LARS are eased when faced with social acceptance [20, 21]. Patients described the feeling of taboo around the TAI equipment and the consciousness of its use. Although, the patients also experienced a sense of relief when having open discussions about TAI with family, friends and colleagues [22]. TAI became a new habit that both the patients and the surroundings adapted to and that consequently eliminated the sense of stigma and embarrassment. Patients described the use of TAI as part of a daily habit, like brushing their teeth or knotting their shoes. Some patients stopped seeing TAI as a treatment but as a part of their lives. As a result, the emotional well-being of the patients increased over time, which otherwise was deeply influenced by bowel dysfunction and loss of control. *Maalouf et al.* reported that patients with LARS had increased anxiety and emotional stress due to bowel dysfunction and restricted leisure. Patients felt embarrassment and vulnerability, causing more isolation [23]. This could be underlined by how the patients in the present study described their lives with LARS prior to the introduction of TAI, as a feeling of being constantly “bound to a toilet” or “locked in at home” [24]. In the EORTC-QLQ-C30, the TAI group showed improved QoL compared with the control group, which are in line with the patients´ experiences described in the interviews. However, there were no significant differences after 6-years of follow-up, which may be explained by a lack of power due to the small cohort making smaller changes harder to show or that patients cope with their symptoms. On the other hand, the EORTC-QLQ-C30 questionnaire may not be the best instrument for longitudinal evaluation of QoL in these patients [21]. Maalouf et al. reported that the items presented are not always representable for rectal cancer patients and the questions may be irrelevant for their situation [21].

Another aspect of bowel dysfunction on personal well-being is the physical discomfort of perianal skin damage and irritation due to frequent toilet visits [25]. According to *Burch et al.* these problems can result in anxiety and psychological discomfort [20], which in turn, can lead to isolation. This was shown in the present study as the patients stressed that TAI had relieved them from these issues, including less irritations in the perianal area. Moreover, the analysis indicates that following an introductory phase, the execution became less vexatious and the sensed initial physical discomfort disappeared. The enema-induced risk for bowel perforation has been estimated to 1/50 000 irrigations in patients with neurogenic bowel dysfunction. For patients with LARS there is no available data estimating the risk for perforation [26].

One of the strengths of the present study is the mean 6-years follow-up of the entire cohort in a randomized trial of two treatment options for LARS. All patients in the control group were invited to try TAI after the end of the RCT. Surprisingly, only two patients from the control group chose to try TAI after study exit and we hypothesize that this low number may partly be explained by the COVID-19 pandemic, which greatly restricted hospital visits at the time. Moreover, an introduction to TAI calls for a clear explanation in a sensitive manner from skilled professionals, which often require hospital visits. Another strength is the high response rate within the TAI group, which could indicate that patients felt that their participation was important and that they wanted to convey their experiences, both negative and positive.

Limitations of the study include the small cohort size in the quantitative analysis, due to dropout. This could mostly have been important for the analysis of QoL where the differences may be smaller. Although the results and discussion points are consistent with current findings regarding LARS and patient experiences with these difficulties. In the present study there is no randomization between the two groups (TAI and control) as in the previous RCT. However, most of the patients remained in the same groups at follow-up, only two patients had tried TAI after the randomization ended. The other patients reported using either TAI or medications to regulate bowel function.

## Conclusions

This long-term follow-up study showed that patients with the TAI treatment reported lower LARS scores after 6-years of follow-up. Patients reported that the use of TAI resulted in regular controlled bowel emptying and the initial experienced discomfort, pain and psychological distress decreased over time. Performing TAI was time-consuming and required careful planning and organization in daily life. Patients who continued with TAI experienced great improvements regarding freedom and participation in social activities due to regained self-control, making the efforts of TAI worthwhile.

## Electronic supplementary material

Below is the link to the electronic supplementary material.

Supplementary Material 1

## Data Availability

The datasets used during the current study are available from the corresponding author on reasonable request.
