# Supplementary material for: The patient perspective on transanal irrigation treatment for low anterior resection syndrome after rectal cancer surgery - a qualitative and quantitative study
Source: BMC Gastroenterol. 2025 Feb 7;25:64. doi: 10.1186/s12876-025-03633-4 (PMC11804026; doi:10.1186/s12876-025-03633-4)
Supplement: Supplementary file 1 — Supplementary Material 1 [file 12876_2025_3633_MOESM1_ESM.docx]

**Supplementary Material 1.**

*Interview questions about the Intervention (TAI) treatment:*

1. What was your general experience of the TAI treatment?
2. Do you have any positive experiences with the TAI treatment that you would like to share?
3. Do you have any negative experiences with the TAI treatment that you would like to share
   1. What difference has the TAI treatment made in your daily life?
4. What was the reason that you continued to use the TAI treatment after the previous study ended?
5. What was the reason that you stopped using the TAI treatment after the previous study ended?
6. What was your experiences with the education that you received before the start of the TAI treatment?
   1. How did you think it was to practically perform the TAI treatment?
7. Is there something you wish to have known before the start of the TAI treatment?
8. Is there something you would recommend us to inform to new patients who would start the TAI treatment?
9. Is there something else that you would like to add regarding the TAI treatment?
10. If we would have any further questions, could we contact you to ask them?
